# Integrated sensing and communication in an optical fibre

**DOI:** 10.1038/s41377-022-01067-1

**Published:** 2023-01-17

**Authors:** Haijun He, Lin Jiang, Yan Pan, Anlin Yi, Xihua Zou, Wei Pan, Alan E. Willner, Xinyu Fan, Zuyuan He, Lianshan Yan

**Affiliations:** 1 Center for Information Photonics & Communications, School of Information Science and Technology, Southwest Jiaotong University, 611756 Chengdu, Sichuan China; 2 Peng Cheng Laboratory, 518052 Shenzhen, China; 3 Optical Fiber Communications Laboratory, University of Southern California, Los Angeles, CA 90089 USA; 4 State Key Laboratory of Advanced Optical Communication Systems and Networks, Shanghai Jiao Tong University, 200240 Shanghai, China

**Keywords:** Fibre optics and optical communications, Optical sensors

## Abstract

The integration of high-speed optical communication and distributed sensing could bring intelligent functionalities to ubiquitous optical fibre networks, such as urban structure imaging, ocean seismic detection, and safety monitoring of underground embedded pipelines. This work demonstrates a scheme of integrated sensing and communication in an optical fibre (ISAC-OF) using the same wavelength channel for simultaneous data transmission and distributed vibration sensing. The scheme not only extends the intelligent functionality for optical fibre communication system, but also improves its transmission performance. A periodic linear frequency modulation (LFM) light is generated to act as the optical carrier and sensing probe in PAM4 signal transmission and phase-sensitive optical time-domain reflectometry (Φ-OTDR), respectively. After a 24.5 km fibre transmission, the forward PAM4 signal and the carrier-correspondence Rayleigh backscattering signal are detected and demodulated. Experimental results show that the integrated solution achieves better transmission performance (~1.3 dB improvement) and a larger launching power (7 dB enhancement) at a 56 Gbit/s bit rate compared to a conventional PAM4 signal transmission. Meanwhile, a 4 m spatial resolution, 4.32-*nε*/$\sqrt {Hz}$ strain resolution, and over 21 kHz frequency response for the vibration sensing are obtained. The proposed solution offers a new path to further explore the potential of existing or future fibre-optic networks by the convergence of data transmission and status sensing. In addition, such a scheme of using shared spectrum in communication and distributed optical fibre sensing may be used to measure non-linear parameters in coherent optical communications, offering possible benefits for data transmission.

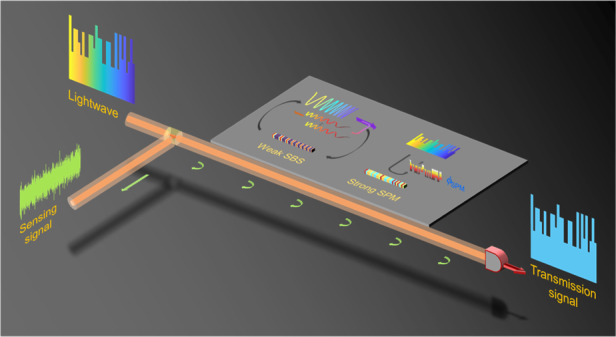

## Abstract (web summary)

A scheme of integrated sensing and communication in an optical fibre (ISAC-OF) using the same wavelength channel for simultaneous high-speed data transmission and distributed vibration sensing.

## Introduction

Over the past few decades, global network traffic has grown explosively due to the demands for higher bandwidth and faster connections of various multimedia and data services (e.g. big data, cloud computing, streaming video, Internet of Things, machine-to-machine communication, and remote surgery)^1,2^. Conventional cable-based transmission approaches cannot meet the enormous data transmission requirements. With the advantages of low loss, large bandwidth, and anti-electromagnetic interference, optical fibres have replaced cables and have been widely used as the transmission medium in fibre to the home (FTTH) networks, metropolitan area networks (MANs), backbone networks, and transoceanic communications^3,4^. Due to the wide application of fibre-optic networks, optical fibres exist anywhere in modern society, and the fibre can be exploited for more functions than data transmission. In addition to acting as the transmission medium in fibre-optic networks, optical fibre can also act as the sensing medium in optical fibre sensors^5–7^. Among the numerous optical fibre sensors, distributed optical fibre sensing (DOFS) interrogates a large number of points in a single sensing fibre, thus providing an overwhelming advantage over conventional node-type sensors in long-distance measurement^6–8^. In particular, DOFS techniques based on backscattered light (including Rayleigh^9–11^, Brillouin^12,13^, and Raman^14^) take into account both the measurement accuracy and sensing distance, hence attracting much attention and intensive study over the last few decades. For these techniques, not only the intrinsic fibre characteristics (length, attenuation, breakpoints^15^, and optical fibre diameter^16^) but also a variety of physical parameters (such as temperature^17^, strain^18^, pressure^19^, seismic wave^20,21^, and refractive index^22,23^) can be measured with high resolution. At present, single-mode fibre (SMF) is a commonly used medium in DOFS (as the sensing medium) and fibre-optic transmission (as the transmission medium). Moreover, various optical communication technologies have been introduced into DOFS to demodulate sensing information and improve sensing performance^24^. The high similarity of DOFS to a fibre-optic communication system in terms of architecture, signal detection, and signal demodulation makes it ideal for integration into a fibre-optic transmission system for simultaneous data transmission and environment monitoring^25^.

Recently, existing optical fibres in fibre-optic networks have been used to measure seismic and water waves based on the laser interference^26^ and state of polarization (SOP) analysis^27–29^. Subsequently, the effect of the geomagnetic field perturbations on the power supply to transoceanic fibre-optic cables has been explored^30^. Using the same sensing mechanisms, integrated schemes of communication and sensing were demonstrated^31,32^. All the above works measure perturbations on the fibre by analyzing the properties of forward transmitted light. These techniques can be easily integrated into the fibre communication system for long range sensing at low cost. However, due to the integral effect over the entire fibre, they are difficult to locate and retrieve all perturbations accurately, especially for chaotic environments^27^. Therefore, these solutions may be suitable for remote transmission scenarios where the measurement environment is stable/quiet and distributed measurements are not necessarily required, such as transoceanic communications. In addition to above techniques, a typical backscattering-based DOFS technique, phase-sensitive optical time-domain reflectometry (Φ-OTDR, which provides the basis for distributed acoustic sensors, DASs), has been successfully applied in many fields, including seismic wave detection (underground^8,21^ and undersea^33,34^), traffic flow monitoring^35^, and geological monitoring^36,37^. Φ-OTDR utilizes the inherent fibre characteristic of Rayleigh backscattering (RBS) to measure dynamic strains (vibrations/intrusions) along the fibre. Although the sensing distance of Φ-OTDR is generally limited to a few tens of kilometres, it offers remarkable advantages of high sensitivity, high accuracy, fast response, and fully distributed measurements. Thus, Φ-OTDR has been used to exploit more functionalities for fibre-optic network^25,33,34,38–41^. Initially, Φ-OTDR only utilized dedicated or idle undersea fibre cables as the sensing medium to detect seismic waves^20,33,34^. In recent years, Φ-OTDR has been integrated into coherent optical communication networks by wavelength-division multiplexing (WDM) and frequency-division multiplexing (FDM) to enable both data transmission and distributed vibration detection^38–41^. With these integrations, traffic monitoring and road roughness detection have already been explored in the existing fibre-optic networks. It is foreseeable that more potential functions such as urban structure imaging, safety monitoring of the city gas pipeline, and ocean detection are also likely to be developed. However, the demonstrated schemes just share fibres in DOFS and fibre-optic networks, and there are still two individual systems. Due to reserving channels for DOFS (fibre or spectrum resources), the current solutions reduce the transmission efficiency. For integrated schemes using individual spectrum channels in communication and DOFS, the transmission performance may be degraded as the strong probe used in DOFS easily excites non-linear effects. Moreover, coherent optical communication is commonly used for long range transmission and seems poorly match to conventional DOFS (which works efficiently in tens of kilometres). Since data transmission is the primary function of fibre-optic networks, an efficient integrated solution should maintain or even improve the transmission performance. Theoretically, it is possible to improve the transmission performance by suppressing or utilizing the non-linear effects^42^. Therefore, the current solutions might not be good choices for implementing integrated sensing and communication in optical fibres, especially for the short/middle range application scenarios.

In this work, we demonstrate a solution for integrating a typical intensity modulation direct detection (IMDD) communication and distributed sensing in an optical fibre to enable simultaneous data transmission and vibration monitoring using the same channel (including the same optical fibre and the same spectrum resources). With periodic single-sideband (SSB) modulation, an LFM optical carrier is generated to carry a 4-level pulse amplitude-modulation (PAM4) signal and acts as a sensing probe to capture vibrations along the fibre. In the experiments, 56 Gbit/s PAM4 signal transmissions and distributed vibration measurements are simultaneously implemented in a 24.5 km fibre. Benefiting from the effective suppression of stimulated Brillouin scattering (SBS) by the LFM optical carrier, the launching power and self-phase modulation (SPM) are significantly enhanced, thereby mitigating chromatic dispersion (CD)-induced power fading and improving the transmission performance. The experimental results show that the optimal launching power is improved by 7 dB in comparison with the conventional PAM4 signal transmission. By employing the respective optimal launching powers in both schemes, the proposed scheme achieves an ~1.3 dB performance improvement at the 7% forward error correction (FEC) threshold (BER = 3.8 × 10^−3^). In the ISAC-OF, only the carrier-corresponding RBS light is detected to retrieve the sensing information. By demodulating the phase, vibrations along a 24.5 km fibre are accurately retrieved with 4 m spatial resolution (SR), 4.32-*nε*/$\sqrt {Hz}$ strain resolution, and an effective sampling rate in excess of 42 kHz. More importantly, the sensing accuracy is only reduced by ~1.25 dB compared with the conventional LFM-based Φ-OTDR, with little impact on sensing performance. Moreover, the effects of transmission light parameters on both transmission and sensing performance are also discussed.

## Results

### Operating principle

**Fig. 1:**
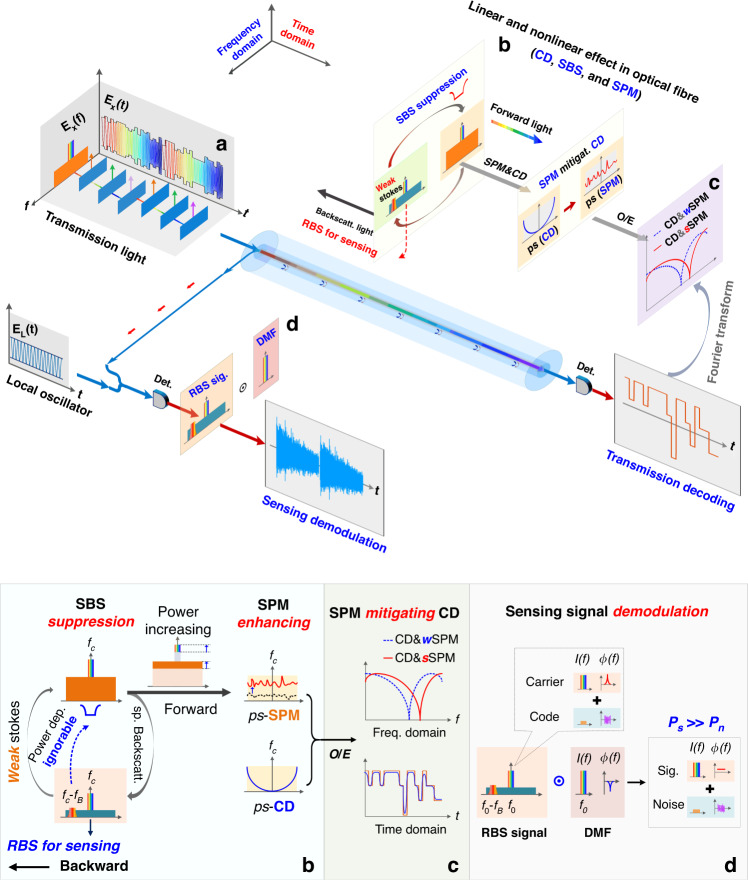
Illustration of the integrated sensing and communication in an optical fibre (ISAC-OF). SBS stimulated Brillouin scattering, SPM self-phase modulation, CD chromatic dispersion, RBS Rayleigh backscattering, *w*SPM weak SPM, *sS*PM strong SPM, Det. detector, DMF digital matched filter, power dep. power depletion, sp. Backscatt. spontaneous backscattering, *ps* phase spectrum. **a** Time and frequency characteristics of the transmission light. **b** The linear and non-linear effects in optical fibre, including light backscattering, CD, SBS, and SPM. **c** The characteristics of the electrical signal for CD mitigation using SPM. The blue dashed line represents the result of the interaction between weak SPM and CD. The red line represents the result of the interaction between strong SPM and CD. **d** The illustration of the sensing signal demodulation in the frequency domain

Figure 1 depicts the operating principle of the proposed ISAC-OF, which is composed of a signal transmitter, fibre link, and signal receivers. In the signal transmitter, an LFM optical carrier is first generated with SSB modulation. Subsequently, the transmission code with PAM4 format is loaded onto the LFM optical carrier to generate the transmission light *E*_*X*_(*t*), as illustrated in Fig. 1a. When the transmission light *E*_*X*_(*t*) is launched into the fibre link, the backscattering light is produced and propagates back to the transmitter. In addition, linear and non-linear effects in the optical fibre interact each other and change the characteristics of the transmission light, especially for a large launching power, as shown in the inset (b). Generally, SBS interaction is detrimental to the transmission performance and it limits the launching power of the transmitted light. In the ISAC-OF, it is hard to construct the steady-state SBS by using an LFM optical carrier, so the power of the backward Stokes light is very small. The power depletion on the optical carrier caused by the weak Stokes can be ignored. This allows the launching power to be further increased, thus dramatically enhancing SPM^43,44^. By elaborately tuning the launching power, an appropriate SPM is produced to mitigate the CD-induced power fading and improve the transmission performance, as illustrated in the inset (c). Meanwhile, the RBS signal is used to retrieve vibrations along the fibre. Since most of the power of the transmitted light *E*_*X*_(*t*) is concentrated on the optical carrier (~95%), only the carrier-corresponding RBS signal is extracted as the sensing signal. In signals detection, two individual receivers are required to detect the transmitted (forward) and sensing (backwards) signals at the remote and local ends respectively. At the remote end, the transmitted light is converted into an electrical signal by direct detection. Due to the unique time-frequency characteristics of the LFM optical carrier, the electrical signal has similar time-frequency characteristics (the same bandwidth) to the one acquired in the conventional PAM4 transmission. Therefore, the same optoelectronic components (photodetector and data acquisition device) and decoding methods are employed as conventional schemes. In the sensing signal demodulation, coherent detection is used to convert the RBS light *E*_*R*_(*t*) at the local end (in the signal transmitter). A digital matched filter (DMF) is generated according to the parameters of the LFM optical carrier and system deployment. By convoluting the DMF and the collected RBS signal, the effective RBS signal is retrieved, as depicted in the inset (d). Although the transmission code introduces additional optical noise to the sensing probe (i.e. the LFM optical carrier), the noise power is much lower than the probe power (*P*_*noise*_≪*P*_*sig*_) and has little impact on the sensing performance.

### Experimental setup

**Fig. 2:**
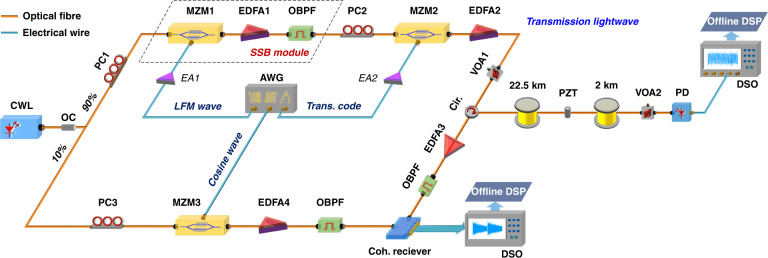
Experimental setup for the integrated sensing and communication in an optical fibre. CWL continuous-wave laser, OC optical coupler, PC polarization controller, MZM Mach-Zehnder modulator, EDFA erbium-doped fibre amplifier, OBPF optical bandpass filter, EA electrical amplifier, AWG arbitrary waveform generator, VOA variable optical attenuator, Cir circulator, PZT piezoelectric transducer, PD photodetector, DSO digital storage oscilloscope, DSP digital signal processing

The experimental setup of the ISAC-OF is shown in Fig. 2. A continuous-wave laser (CWL) with ultra-narrow linewidth (NKT Koheras Basik X15, linewidth <0.1 kHz) acts as the laser source. The centre wavelength and the output power of the laser are 1549.5 nm and 13 dBm, respectively. The CW light is split into two branches by a 90:10 optical coupler (OC). The light in the upper branch (90%) is first modulated to generate an LFM optical carrier with SSB modulation. The SSB modulation module includes a Mach-Zehnder modulator (MZM, iXblue MX-LN-20), an erbium-doped fibre amplifier (EDFA, Amonics AEDFA-PA-35-B-FA), and a tuneable optical bandpass filter (OBPF, EXFO XTM-50). Driven by the electrical LFM waveform and operating at the minimum transmission point, MZM1 implements double sideband suppressed carrier modulation (DSB-SC). To compensate for the insertion loss, the EDFA1 is used to boost the power of the modulated light to 11 dBm. The OBPF is used to remove the -1st order sideband and the residual optical carrier, thereby achieving an LFM optical carrier with constant power. Here, the LFM waveform is generated by an arbitrary waveform generator (AWG, Keysight M8195A with four channels) with a bandwidth and maximum sample rate of 25 GHz and 65 GSa/s, respectively. To accurately demodulate the sensing information, the repetition period of the LFM waveform should be larger than the time of light travelling through the entire fibre^45^. A 24.5 km fibre (~245 us) is used in the experiments and we fix the repetition period of the LFM waveform to 248 us, corresponding to a distance of 24.8 km, slightly longer than the fibre length. After generating the LFM optical carrier, the transmission code is modulated onto the optical carrier by another MZM (MZM2, Sumitomo T.MXH1.5-40PD-ADC). Similar to conventional PAM4 signal transmission, MZM2 operates in the linear region. In the message generation part, a test sequence with a 2^15^-1 pseudorandom bit sequence (PRBS) is generated and mapped into PAM4 with 2 samples per symbol. The upsampled signals are shaped using a square root raised cosine (SRRC) with a roll-off factor of 0.1. Next, a pre-distortion operation is utilized to overcome the frequency roll-off of the digital-to-analogue converter (DAC). Before being injected into the 24.5 km SMF, the transmission light is enhanced by an EDFA (EDFA2) with 18 dBm output power, and the launching power is adjusted by a variable optical attenuator (VOA). To validate the sensing capability, a piezoelectric transducer (PZT) is inserted at the 22.5 km position to produce dynamic strains. At the receiving end, the power of the transmitted light is first tuned by a VOA and then directly detected with a photodetector (PD, Agilent 11,982 A with a 3 dB bandwidth of 15 GHz). The output electrical signal of the PD is sampled by a digital storage oscilloscope (DSO, Keysight DSOZ634A) with a 25 GHz bandwidth and an 80 GSa/s sampling rate.

In the lower branch, 3 dBm CW light (10%) is modulated by MZM3 (Sumitomo T.MXH1.5-40PD-ADC) to generate a local oscillator (LO). MZM3 works at the minimum transmission point and is driven by a standard cosine waveform at 9.8 GHz. To acquire good detection performance, an EDFA (Amonics AEDFA-PA-35-B-FA) and an OBPF (Finisar Waveshaper 4000 s) are used to amplify the LO and eliminate redundant sidebands, respectively. At the transmitting terminal, the amplified RBS light is combined with the LO and then converted into an electrical signal at a coherent receiver (Finisar CPVR1225A, 25 GHz bandwidth). It should be noted that the use of the coherent receiver eliminates polarization fading in coherent-based Ф-OTDR and improves the robustness of the integrated solution. Finally, the electrical signal is sampled by a 4 GHz DSO (LeCroy Waverunner 8404 M) and processed offline.

### Transmission performance

**Fig. 3:**
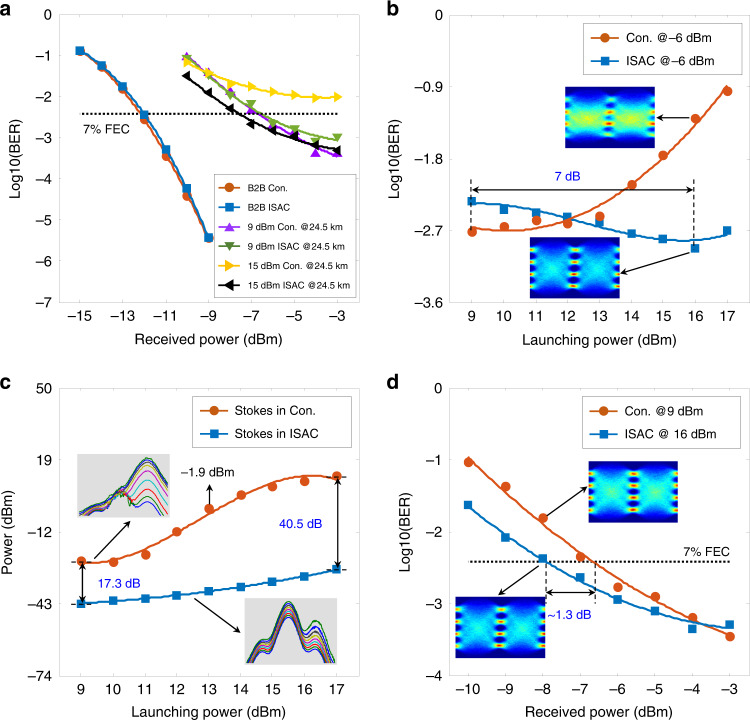
Comparison of the BER performance in the conventional PAM4 signal transmission (Con.) and integrated sensing and communication in optical fibre (ISAC). **a** BER performance of 56 Gbit/s PAM4 signal in B2B and 24.5 km fibre transmission. The results for 24.5 km fibre transmission using 9 dBm and 15 dBm launching power are shown here. **b** BER performance evolution of 56 Gbit/s PAM4 signal as the launching power increases. The received power was fixed at −6 dBm in all tests. **c** Power evolution of the backwards Stokes light as the launching power increases. **d** BER performance of 56 Gbit/s PAM4 signal in Con. (9 dBm launching power) and ISAC (16 dBm launching power) for different received powers

The transmission performance of the proposed ISAC-OF has been measured under different launching powers, received powers, and LFM bandwidths. For comparison, the conventional single-carrier 56 Gbit/s PAM4 transmission is also performed under back-to-back (B2B) and 24.5 km conditions. Figure 3a shows the BER evolution with the increment of the received power. Impacted by SSB modulation, the 1-GHz LFM optical carrier has a worse optical signal-to-noise ratio (OSNR) than the single-frequency optical carrier (i.e., the laser source used in conventional schemes). Thus, the integrated scheme (the blue-dotted line) has an approximately 0.15-dB power penalty (an ignorable performance degradation) at the 7% FEC threshold in comparison with the conventional scheme (the orange-dotted line). In fibre transmission, the CD-induced power fading deteriorates the BER performance. By adopting a 9 dBm launching power, both schemes achieve almost the same BER performance at the 7% FEC threshold (the purple and green lines), as illustrated in Fig. 3a. Compared to the B2B BER performance, there is an ~5 dB power penalty after 24.5 km fibre transmission. Moreover, the BER performance using 15 dBm launching power is also measured and depicted in Fig. 3a. Affected by the strong SBS, the BER performance of the conventional scheme (the orange-dotted line) deteriorates significantly compared to the results measured using a 9 dBm launching power (the purple-dotted line). In the ISAC-OF, the LFM optical carrier suppresses the SBS interaction between the optical carrier and the backwards Stokes light, thus eliminating the detrimental effect of SBS on the transmission performance. Moreover, the SPM effect is enhanced as the launching power is increased to 15 dBm, which mitigates the CD-induced power fading. Compared to the results measured with a 9 dBm launching power in both schemes (the green- and purple-dotted lines), a 1 dB BER performance improvement is achieved in the integrated scheme using a 15 dBm launching power (the black-dotted line). In the ISAC-OF, the PAM4 transmission performance is codetermined by CD, SBS, and SPM effects. Since both SBS and SPM effects depend on the launching power, the BER evolution is measured by increasing the launching power, as depicted in Fig. 3b. In the experiments, we fix the received power at −6 dBm and increase the launching power from 9 to 17 dBm with a step of 1 dB. In the conventional scheme, a strong SBS interaction is easily constructed between the single-frequency optical carrier and the generated backwards Stokes light. As the launching power increases, the power of the Stokes light and the corresponding power depletion on the optical carrier increase dramatically, hence distorting the transmission code and deteriorating the BER performance, as the orange-dotted line shows in Fig. 3b. To thoroughly analyze the relationship between the launching power and the BER performance, the backwards Stokes power is also measured, as illustrated in Fig. 3c. As the launching power increases, the Stoke power (the orange-dotted line) improves over 46 dB. Here, the insets in Fig. 3c depict the evolution of the optical spectra. When the launching power is less than 13 dBm, the Stokes power is not more than −1.9 dBm, such that the power depletion on the optical carrier can be ignored. Accordingly, the BER performance fluctuates slightly, as shown in Fig. 3b. With a further increase in the launching power (over 13 dBm), the enhanced Stokes light dramatically depletes the optical carrier, hence significantly deteriorating the BER performance. The Stokes power reaches 12 dBm when the launching power is increased to 17 dBm. Different from the conventional scheme, an LFM optical carrier is used in ISAC-OF, and it effectively suppresses the SBS interaction. As shown in Fig. 3c, the Stokes power increases slowly with increasing launching power (the blue-dotted line), and the maximum Stokes power is only −28.2 dBm (much less than −1.9 dBm). Thus, the SBS-induced power depletion on the optical carrier is negligible, and there should be no BER performance degradation. In addition to the SBS effect, the SPM effect is also enhanced with increasing launching power. Furthermore, an appropriate SPM effect mitigates the CD-induced power fading and improves BER performance^43,44^. As depicted in Fig. 3b, the BER performance is first improved and then reduced as the launching power increases (the blue-dotted line). The optimal BER performance is achieved at a 16 dBm launching power, an ~7 dB improvement in launching power compared to the conventional scheme (the orange-dotted line, the optimal launching power is 9 dBm). To accurately analyze the BER performance improvement, the launching power of the conventional scheme and the integrated scheme are fixed at 9 and 16 dBm, respectively. By tuning the received power, the BER performance is measured and shown in Fig. 3d. Compared to the conventional scheme (the orange-dotted line), the integrated scheme (the blue-dotted line) achieves an ~1.3 dB improvement at the 7% FEC threshold. Benefitting from the larger launching power and better BER performance, the ISAC-OF can provide better transmission performance in fibre-optic networks, such as MAN and passive optical network (PON).

**Fig. 4:**
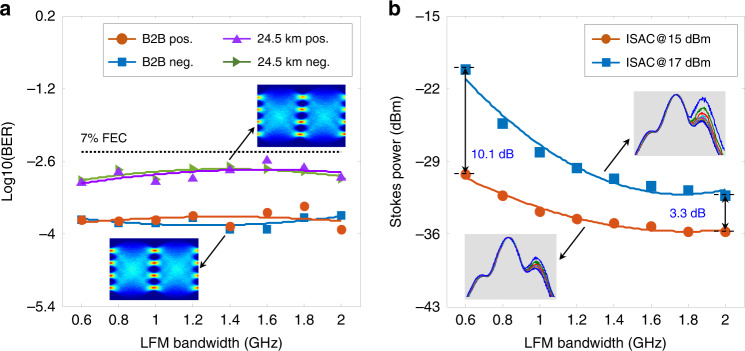
The effect of LFM bandwidth on the BER performance. **a** BER performance of the PAM4 signal using positive (pos.) and negative (neg.) LFM optical carrier in the B2B test and 24.5 km fibre transmission. **b** The power evolution of the backwards Stokes light with the increment of the LFM bandwidth. Here two different launching powers of 15 dBm and 17 dBm were tested

In principle, SBS can be suppressed more thoroughly by employing a larger bandwidth LFM optical carrier. To verify this property and analyze the effect of bandwidth on the transmission performance, we conduct experiments using LFM optical carriers with different bandwidths (including positive and negative). In these experiments, a 56 Gbit/s PAM4 signal is utilized as the transmission signal, and the LFM bandwidth is increased from 0.6 to 2 GHz in 0.2 GHz steps. In the B2B tests, the received power is fixed at −11 dBm, and the BER performance is illustrated in Fig. 4a. Except for slight fluctuations, the BER performance remains almost unchanged with increasing LFM bandwidth. Moreover, the positive LFM optical carrier achieves nearly the same BER performance as the negative one. In the 24.5 km fibre transmission, the launching power and the received power are fixed at 15 and −6 dBm, respectively. The BER performance (the purple and green lines) remains constant for all bandwidths. Meanwhile, the Stokes power is detected, and the power decreases with increasing bandwidth, as shown in Fig. 4b. This means that the LFM optical carrier with a larger bandwidth suppresses the SBS effect more thoroughly. For a 15 dBm launching power (the orange-dotted line), the Stokes power is too small for all bandwidths (the maximum power is only −30 dBm), and the corresponding power depletion on the optical carrier can be ignored. Hence, the test bandwidth has little effect on the BER performance, as shown in Fig. 4a. Subsequently, the launching power is improved to 17 dBm, and the Stokes power at different bandwidths is measured, as shown by the blue-dotted line in Fig. 4b. Compared to the results using a 15 dBm launching power, the Stokes power is increased by more than 10 dB on the 0.6 GHz optical carrier. As the carrier bandwidth increases to 1.2 GHz, the Stokes power drops to almost the same value as when using a 15 dBm launching power. Thus, a large-bandwidth LFM optical carrier is required to maintain the BER performance when a large launching power is used.

### Sensing results

**Fig. 5:**
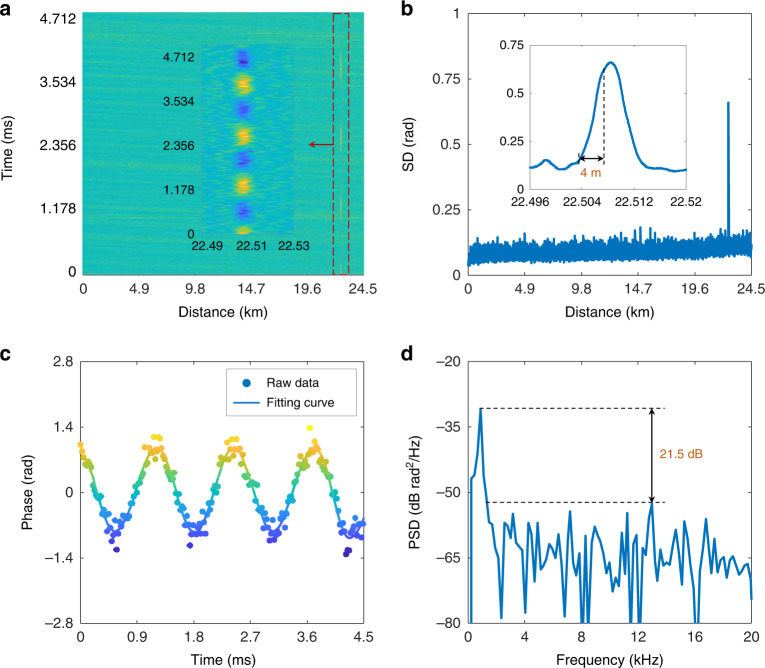
Time and frequency characteristics of the measured vibration. **a** The distance–time distribution of the differential phases along the fibre, with a vibration applied at around 22.5 km. **b** Phase SD along the fibre with a spatial resolution of ~4 m. **c** Time-domain waveform and **d** corresponding PSD of the measured vibration

In addition to the transmission performance, the sensing performance of the ISAC-OF is also tested. A 56 Gbit/s PAM4 signal is first loaded on a 1 GHz LFM optical carrier, and the transmission light with 15 dBm launching power is launched into the fibre. In the experiment, a 5 m bare fibre is coiled around a PZT at a 22.5 km location. A sinusoidal waveform with an 800 Hz frequency and a 0.6 V amplitude is used to actuate the PZT. The detected RBS signal is collected by a DSO with a sampling rate of 2.5 GSa/s. By convoluting the collected RBS signal with the corresponding DMF, the effective RBS signal is demodulated. In the phase retrieval, the interference fading is eliminated with the phase shift transform and the rotated-vector-based moving average^46–48^. Here, the rotated-vector-based moving average is performed along the distance axis. In Φ-OTDR, the intrinsic frequency response is limited by the probe repetition rate, which is equal to 2.016 kHz in this work. Benefitting from the time-frequency characteristics of the continuous periodic LFM probe (i.e. the LFM optical carrier), the frequency response of the integrated scheme can be significantly improved by signal segmentation and timing splicing^45,49^. Moreover, the enhancement factor (EF) of the frequency response can be flexibly adjusted in signal processing. But it must be noted that the frequency response enhancement is achieved by sacrificing the spatial resolution (SR) for the same signal. In the signal segmentation, the bandwidth of the subband signal (*B*_*s*_) is reduced with increasing EF, and *B*_*s*_ is equal to *B*/EF (*B* is the LFM bandwidth). Since the effective SR is codetermined by the bandwidth of the subband signal, averaging distance, and gauge length (GL), the increment of the frequency response may reduce the effective SR. To balance the SR and frequency response, EF and the average distance are set to 10 (20.16 kHz frequency response) and 4 m, respectively. With a 4 m GL, the distance–time distribution of differential phases is calculated, and the top view is depicted in Fig. 5a. The vibration can be clearly discerned, and there is no erroneous phase, indicating that the interference fading is eliminated well. By calculating the standard deviation (SD) of the differential phases, the effective SR is shown in Fig. 5b. With the conventional definition of the length between 10 and 90% rising edge, a 4 m SR is achieved. Note that, for the same sensing signal, a higher SR can be achieved by reducing EF in the signal processing. Figure 5c depicts the time-domain waveform with a peak-to-peak phase of ~1.87 *rad*, corresponding to a peak-to-peak strain of 51.58 *nε*^50^. The effective sample rate is increased by 10 times, and the frequency response exceeds 20 kHz. The corresponding power spectrum density (PSD) is calculated, and a signal-to-noise ratio (SNR) of 21.55 dB is obtained, as illustrated in Fig. 5d. The maximum noise level is ~−52.55 dB *rad*^2^/*Hz*, so the worst strain resolution is ~4.32-*nε*/$\sqrt {Hz}$.

**Fig. 6:**
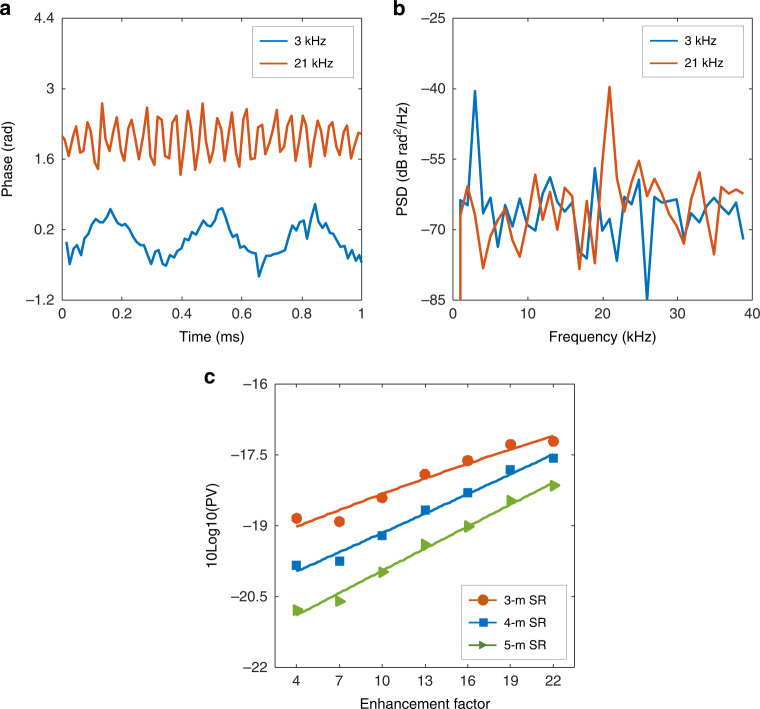
Characteristics of the frequency response enhancement. **a** The time-domain waveforms of 3 kHz and 21 kHz vibrations and **b** the corresponding PSDs. **c** The effect of enhancement factor (EF) on the sensing accuracy with different spatial resolutions (SRs)

To verify the effectiveness of the frequency response enhancement, vibrations with driving frequencies of 3 kHz and 21 kHz are applied to the fibre. Here, both driving frequencies exceed the intrinsic 2.016 kHz frequency response, and both driving voltages are 0.3 V. The bandwidth of the LFM optical carrier, bit rate of the PAM4 signal, and launching power of the transmission light are 1 GHz, 56 Gbit/s, and 15 dBm, respectively. In the signal demodulation, the averaging distance, EF, and GL are set to 5 m, 20 (the maximum frequency response is 40.32 kHz), and 5 m, respectively. Figure 6a and b depicts the time-domain waveforms and corresponding PSD, respectively. Both vibrations are accurately retrieved, indicating the effectiveness of the frequency response enhancement. It must be noted that the sensing accuracy theoretically varies as the frequency response increases. If the same SR (same averaging distance and GL) is used in the signal demodulation, a large-bandwidth RBS signal can eliminate the interference fading more completely and achieve better sensing accuracy. However, the sensing accuracy is also related to the duration of the subband signal due to the impact of the laser phase noise. Moreover, the sensing accuracy decreases with the duration of the subband signal^51^. As EF increases, both bandwidth and duration of the subband signal decrease, so there is a trade-off between eliminating interference fading and laser-induced phase noise (which determines the sensing accuracy). To clarify the effect of the frequency response enhancement on the sensing accuracy, RBS signals with a 1 GHz bandwidth are demodulated with different enhancement factors. In the signal demodulation, EF is increased from 4 to 22 in 3 steps, and both the average distance and GL are fixed at 4 m (corresponding to a 4 m SR). Since the phase variance (PV) calculated with the signal without vibration accurately represents the phase noise floor, we calculate the PV at ~22.5 km and transform its units with the expression of 10log10(PV), as shown by the blue-dotted line in Fig. 6c. With the increment of EF, the PV is increased by 2.63 dB (corresponding to a 2.63-dB reduction in sensing accuracy). Here, the bandwidth of the subband signal is reduced from 250 MHz down to 45.5 MHz. The experimental results show that the larger bandwidth achieves better sensing accuracy, indicating that the sensing accuracy is mainly dominated by the eliminating performance of the interference fading. Moreover, the same signals are processed with 3 m (3 m average distance and 3 m GL, the orange-dotted line) and 5 m SR (5 m average distance and 5 m GL, the green-dotted line), respectively. Compared to the results demodulated with 4 m SR, the sensing accuracy of demodulation with 3 m SR and 5 m SR is reduced and improved by 0.71 and 0.75 dB, respectively.

**Fig. 7:**
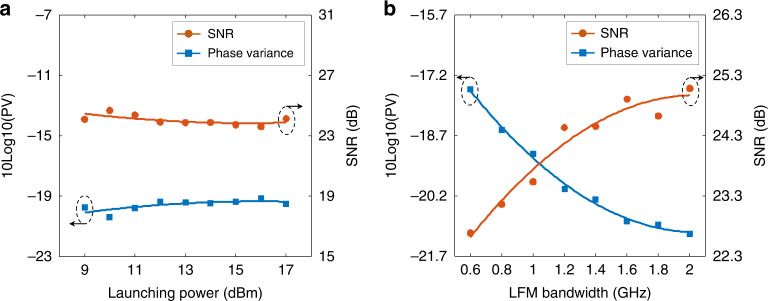
Effect of the parameters of the transmitted light on the sensing performance. **a** The effect of the launching power, which is increased from 9 to 17 dBm in a step of 1 dB. **b** The effect of the LFM bandwidth, which is increased from 0.6 to 2 GHz in steps of 0.2 GHz

Subsequently, the effects of the launching power and the LFM bandwidth on the sensing performance are tested. In the launching power test, a 56 Gbit/s PAM4 signal is loaded on the 1 GHz optical carrier. The launching power of the transmission light is increased from 9 to 17 dBm with a step of 1 dB. The collected RBS signals are demodulated with 10 EF, 4 m average distance, and 4 m GL. Figure 7a depicts the SNR and the corresponding PV. Since the power of the effective RBS light (generated by the LFM optical carrier) increases synchronously with the power of the code-induced noise, both the SNR (the orange-dotted line) and PV (the blue-dotted line) remain almost constant for all launching powers. This indicates that the sensing accuracy is independent of the launching power.

As mentioned above, a large-bandwidth signal provides better elimination of interference fading and acquires better sensing accuracy. However, it must be noted that in the EF effect analysis, as shown in Fig. 6c, the SNR of the subband signals remains almost the same although the bandwidth changes. Actually, the sensing accuracy is related not only to the eliminating performance of the interference fading but also to the SNR of the RBS signal. In principle, an RBS signal with a larger LFM bandwidth has a smaller SNR if the launching power remains unchanged. Therefore, we discuss the effect of the LFM bandwidth on the sensing accuracy. In the experiments, a 56 Gbit/s PAM4 signal is loaded on a 1 GHz LFM optical carrier, and the launching power is fixed at 15 dBm. The LFM bandwidth of the optical carrier is increased from 0.6 to 2 GHz with a step of 0.2 GHz. When the signal bandwidth is less than 1 GHz, the RBS signal is collected at a sampling rate of 2.5 GSa/s. Otherwise, the RBS signal is sampled by a 5 GSa/s sample rate. For all signals, a 10-fold frequency response enhancement (EF = 10), a 4 m average distance, and a 4 m GL are used in the signal demodulation. With the increment of the LFM bandwidth, the SNR is increased by 2.4 dB, as shown by the red-dotted line in Fig. 7b. Accordingly, the phase variance is reduced by 3.59 dB (the blue-dotted line), corresponding to a 3.59 dB improvement in sensing accuracy. The performance improvement indicates that the sensing accuracy is mainly dominated by the carrier bandwidth.

**Fig. 8:**
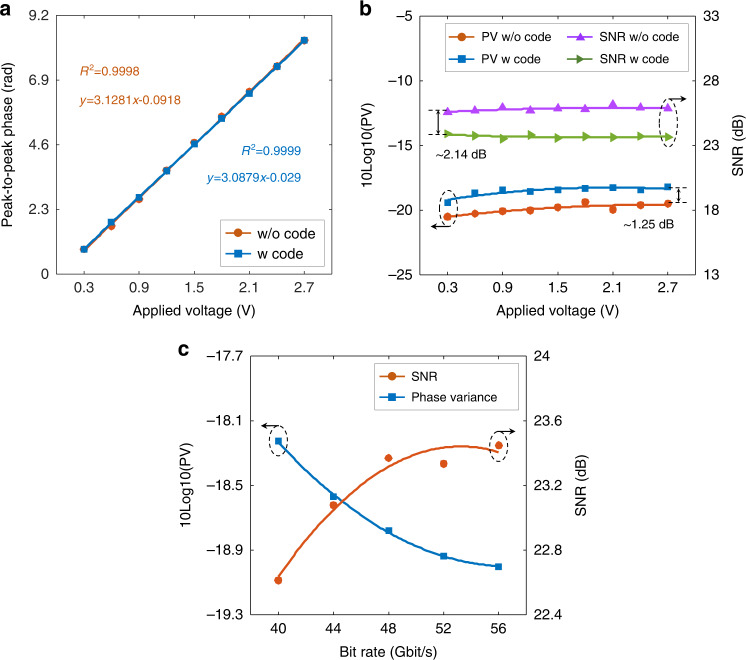
The effect of the transmission code on the sensing performance in the ISAC-OF. **a** Comparison of the sensor response using a pure LFM optical probe (w/o code) and ISAC-OF (w code). **b** Comparison of the sensing accuracy (which is determined by PV) and SNR in the conventional LFM-based Φ-OTDR and ISAC-OF. **c** The effect of the bit rate of the transmission code on the sensing accuracy

Finally, the effect of the transmission code on the sensing performance is evaluated. First, a pure LFM optical carrier with a 1 GHz bandwidth and 15 dBm launching power is launched into the fibre. Meanwhile, a vibration driven by a sinusoidal waveform (with a frequency of 800 Hz) is applied to the fibre via a PZT at the 22.5 km position. The sensor response to the vibration intensity is calibrated by improving the driving voltage from 0.3 to 2.7 V with a step of 0.3 V. In the signal demodulation, EF, average distance, and GL are set to 10, 4 m, and 4 m, respectively. The peak-to-peak phase is calculated, and the results are shown by the orange-dotted line in Fig. 8a. The phase value is linearly proportional to the applied voltage (which corresponds to the vibration amplitude) with a linearity of 0.9998. After that, a 56 Gbit/s PAM4 signal is loaded onto the LFM optical carrier, and the launching power still remains at 15 dBm. The peak-to-peak phase is calculated and depicted as the blue-dotted line in Fig. 8a. It must be noted that the transmission code remains the same as that used in the transmission performance verification (including the code format and signal power). Compared to the results without transmission code (the orange-dotted line), the phase response to the vibration amplitude is nearly identical, and the linearity is equal to 0.9999, indicating that the code loading does not distort the sensor response. Furthermore, the SNR and phase variance are computed to estimate the performance degradation, as illustrated in Fig. 8b. Compared with the SNR using the pure LFM optical carrier (the purple line), there is approximately a 2.14 dB SNR degradation when the transmission code is loaded (the green line). Accordingly, the phase variance is increased by 1.25 dB, slightly deteriorating the sensing accuracy. As described in the operation principle, only the carrier-corresponding RBS light is utilized to demodulate the perturbation along the fibre. The noise within the carrier bandwidth is detrimental to the sensing accuracy. For a fixed launching power, the code-induced noise power within the carrier bandwidth decreases as the bit rate increases. To verify this characteristic, we increase the bit rate from 40 to 56 Gbit/s with a step of 4 Gbit/s. In this operation, the LFM bandwidth and the launching power are fixed at 1 GHz and 15 dBm, respectively. Figure 8c depicts the demodulated results, and the SNR of the RBS signal improves with increasing bit rate. Compared with the results of transmitting a 40 Gbit/s signal, the SNR and sensing accuracy of transmitting a 56 Gbit/s signal are improved by 0.83 and 0.77 dB, respectively. The experimental results show that the larger bandwidth transmission signal has less impact on the sensing accuracy.

The above experimental results show that a higher spatial resolution, higher frequency response, and better measurement accuracy can be simultaneously achieved using a large-bandwidth LFM optical carrier. Moreover, the larger bandwidth LFM optical carrier suppresses SBS more effectively, which ensures that a larger launching power can be used in the ISAC-OF. Compared to the integrated IMDD communication and distributed sensing schemes based on WDM/FDM mechanism, the proposed scheme has higher spectral efficiency, lower demodulation complexity (in transmission), and better transmission performance. Therefore, the proposed scheme may be a better choice for integrated transmission and sensing in fibre.

## Discussion

### Applicability

This work proposes an integrated sensing and communication solution for simultaneous PAM4 signal transmission and distributed vibration measurements. Although only the PAM4 signal is performed in the experiments, this scheme is also applicable for other amplitude-modulation-based signals, such as OOK, PAM6, and PAM8 signals. For these signal formats, the LFM carrier still provides the benefits of SBS suppression and SPM enhancement, resulting in better transmission performance than the transmission-only schemes. The sensing performance of systems using PAM formats improves as the number of modulation level increases. For the integrated system using the OOK format, excellent transmission and sensing performance can be simultaneously obtained by adjusting the signal amplitude. Based on the concept of shared spectrum, the periodic LFM waveform can also be integrated into coherent optical transmission for long range transmission and sensing using new modulation and demodulation methods. Theoretically, the LFM waveform embedded in the transmission signal can be eliminated by signal synchronization, thus restoring accurate transmission signals.

### Spectral efficiency, demodulation complexity, and transmission performance

In recent years, several works have integrated coherent optical communication and DOFS using FDM and WDM^25,38–41^. Generally, coherent optical communication is used for long range transmission. In short/middle range communications, IMDD techniques are more commonly used. In an integrated IMDD communication and DOFS system, individual spectrum resources are required for data transmission and DOFS (shared fibre only), which reduces the spectral efficiency. Moreover, the individual sensing probe needs to be eliminated in transmission decoding, which increases the decoding complexity and may degrade transmission performance. In the proposed ISAC-OF, an LFM optical carrier is used to carry the transmission code and simultaneously act as the sensing probe. The transmitted light has the same bandwidth as the transmitted data in a short time. In particular, The LFM waveform is modulated to the phase of the optical carrier, but the transmission signal is loaded to the amplitude. With direct detection, only the signal amplitude is preserved, while the signal phase is omitted. As a result, the electrical signal detected in this solution has the same bandwidth as the one in the conventional PAM4 signal transmission. Benefitting from this property, the proposed scheme uses the same detection components and demodulation methods as the conventional PAM4 signal transmission. More importantly, the introduction of the LFM optical carrier improves the transmission performance. Based on the above analysis, the proposed integration scheme has higher spectral efficiency, lower demodulation complexity in transmission, and better transmission performance compared with an integrated IMDD communication and sensing system based on WDM/FDM.

### System simplification and performance improvement

Restricted by the devices currently used in the experimental setup, the system is complicated. Actually, the detection of the sensing signal can be significantly simplified by using appropriate devices. For instance, if a dedicated optical filter with a narrow filter edge is used to produce the LFM optical carrier. The frequency offset can be greatly reduced and the local light (in the lower branch) can be replaced by the laser source. In addition, the LFM optical carrier can be directly used as the local light based on dechirp operation^52^. In this work, the power of the RBS light is sufficiently large that amplification and filtering can be removed if appropriate detectors are used^45,47,50^. Although the optical amplification used in SSB modulation improves the carrier power, it also reduces the OSNR of the optical carrier. Since both transmission and sensing performance are dominated by OSNR, it is critical to improve the carrier’s OSNR. Optical injection locking can enable high-performance SSB modulation and may be an alternative for generating a high-performance LFM optical carrier^19,53,54^.

### The potential function of the integrated sensing and communication in optical fibre

Rayleigh scattering is an elastic scattering, and the variations in the forward light (time and frequency characteristics) will be fed back to the RBS light. Hence, the forward light variations caused by non-linear effects can be theoretically analyzed with the corresponding RBS light. Using the analyzed non-linear parameters, the signal distortion induced by the non-linear effects might be corrected, thereby improving the transmission performance.

## Materials and methods

### LFM optical carrier generation

To obtain excellent transmission performance, the amplitude of the LFM optical carrier should be kept as constant as possible. In practice, the amplitude of the LFM optical carrier may change due to the variation of the RF power, especially at the frequency burst point. Such a case, a pre-distortion can be implemented in the LFM waveform generation to compensate for the amplitude fluctuations of the LFM optical carrier.

### Transmission decoding

In this work, the same decoding approach as the conventional PAM4 signal transmission is used to decode the transmission signal^55^. The decoding algorithm includes five steps: (1) Resample, the signals are resampled to 4 Sa/symbol. (2) Matched filtering, the signals are processed by a matched filter to recover the effective signals. (3) Clock recovery and down-sampling, the Gardner’s method is used to recover the clock and get the optimum sampling point^56^, and then downsample the signals to 1 Sa/symbol for further processing. (4) Equalization, the channel equalization is implemented by the decision-directed least-mean-square (DD-LMS) error algorithm. (5) Decision and BER calculation.

### Sensing signal demodulation

The orthogonal RBS signals collected from the coherent receiver are first converted into complex signals in the digital domain. By convoluting the complex RBS signals with the corresponding DMF, the effective RBS signals are obtained. The DMF *s*_*DMF*_(*t*) is generated in the digital domain based on the DC component of the LFM optical carrier, which is expressed as.

1 $$s_{{\mathrm{DMF}}}\left( t \right) = \exp \left[ {j2\pi \left( {f_0 - f_1} \right)t + j\pi kt^2 + j\frac{\pi }{4}} \right]$$

where *f*_0_ and *f*_1_ denote the initial frequency of the LFM optical carrier and the frequency of the local light, respectively. *k* is the chirp rate of the LFM waveform, equal to *B*/*T*_*p*_, *B* and *T*_*p*_ are the bandwidth and repetition period of the LFM optical carrier, respectively. The intrinsic spatial resolution is determined by the bandwidth of LFM waveform and equal to *c*/(2*n*_*g*_*B*), where *c* is the light velocity in vacuum, *n*_*g*_ is the effective refractive of the fibre.

Due to the time-frequency characteristics of the continuous periodic LFM optical carrier, the frequency response of the system can be increased by signal segmentation and timing splicing in the signal processing^45,48^. In signal segmentation, the RBS signal is divided equally into EF parts in the frequency domain, and the bandwidth of the subband signal (*B*_*s*_) is equal to *B*/EF. By calculating the differential phases with a predefined GL and splicing the differential phases according to the time series of each subband signal, the effective sensing signals are obtained. Note that EF can be flexibly adjusted in the signal processing. In the phase retrieval, the phase shift transform^46^ and the rotated-vector-based moving average^47,48^ are performed to eliminate the interference fading. Here, the rotated-vector-based moving average is performed along the distance axis.

### SNR calculation

The RBS signals with interference fading elimination are used to calculate the SNR. The RBS signal is a two-dimensional (2D) array and the signal amplitude is denoted as *A*(*z*,*t*). *z* is the fibre location generating the RBS signal, *t* denotes the time at position *z*. The average amplitude *A*(*z*) and amplitude variance *V*[*A*(*z*)] of the RBS signal at position *z* are calculated along the time axis. The signal power *P*_*s*_ and noise power *P*_*n*_ are calculated using the equations *P*_*s*_ = *A*^2^(*z*) and *P*_*n*_ = *V*[*A*(*z*)], respectively. Then, the average signal power and noise power around the vibration location (22.5 km) are calculated as the effective signal power *P* and effective noise power *N*, respectively. Finally, the SNR is computed with the expression 10log10(*P*/*N*).

2 $${\mathrm{SNR}} = 10\log 10\left( {\frac{{\mathop {\sum}\limits_{z_i} {\left\{ {{\mathrm{mean}}\left[ {A\left( {z_i,t} \right)} \right]} \right\}^2} }}{{\mathop {\sum}\limits_{z_i} {{\mathop{{{\rm{var}}}}} \left[ {A\left( {z_i,t} \right)} \right]} }}} \right)$$

where *mean*[] and var[] denote the average and variance operators, respectively. *z*_*i*_ is the fibre location that generates the RBS signal.

### Phase variance calculation

The phase is first retrieved using the RBS signals with interference fading elimination. The phase array is denoted as *φ*(*z*, *t*) and the phase variance *V*[*φ*(*z*)] is calculated along the time axis. The effective phase variance is calculated by averaging the phase variances around the vibration position (22.5 km), which accurately represents the phase noise floor and sensing accuracy.

## Supplementary information

Supplementary Information for Integrated Sensing and Communication in an Optical Fibre

## References

[1] Al-Turjman F, Ever E, Zahmatkesh H (2019). Small cells in the forthcoming 5G/IoT: traffic modelling and deployment overview. IEEE Commun. Surv. Tut..

[2] Essiambre RJ (2010). Capacity limits of optical fiber networks. J. Lightwave Technol..

[3] Liu X (2022). Enabling optical network technologies for 5G and beyond. J. Lightwave Technol..

[4] Winzer PJ, Neilson DT, Chraplyvy AR (2018). Fiber-optic transmission and networking: the previous 20 and the next 20 years [Invited]. Opt. Express.

[5] Liu QW, He ZY, Tokunaga T (2015). Sensing the earth crustal deformation with nano-strain resolution fiber-optic sensors. Opt. Express.

[6] Russell SJ, Brady KRC, Dakin JP (2001). Real-time location of multiple time-varying strain disturbances, acting over a 40- km fiber section, using a novel dual-Sagnac interferometer. J. Lightwave Technol..

[7] Rogers A (1999). Distributed optical-fibre sensing. Meas. Sci. Technol..

[8] Jousset P (2018). Dynamic strain determination using fibre-optic cables allows imaging of seismological and structural features. Nat. Commun..

[9] Barnoski MK (1977). Optical time domain reflectometer. Appl. Opt..

[10] Soriano-Amat M (2021). Time-expanded phase-sensitive optical time-domain reflectometry. Light Sci. Appl..

[11] Muñoz F, Soto MA (2022). Enhancing fibre-optic distributed acoustic sensing capabilities with blind near-field array signal processing. Nat. Commun..

[12] Mizuno Y (2016). Ultrahigh-speed distributed Brillouin reflectometry. Light Sci. Appl..

[13] Sun XZ (2020). Genetic-optimised aperiodic code for distributed optical fibre sensors. Nat. Commun..

[14] Li J, Zhang MJ (2022). Physics and applications of Raman distributed optical fiber sensing. Light Sci. Appl..

[15] Aoyama KI, Nakagawa K, Itoh T (1981). Optical time domain reflectometry in a single-mode fiber. IEEE J. Quantum Electron..

[16] Hua ZJ (2021). Non-destructive and distributed measurement of optical fiber diameter with nanometer resolution based on coherent forward stimulated Brillouin scattering. Light. Adv. Manuf..

[17] Soto MA, Ramírez JA, Thévenaz L (2016). Intensifying the response of distributed optical fibre sensors using 2D and 3D image restoration. Nat. Commun..

[18] Zhou DW (2018). Single-shot BOTDA based on an optical chirp chain probe wave for distributed ultrafast measurement. Light Sci. Appl..

[19] Qiu LQ (2022). High-sensitivity dynamic distributed pressure sensing with frequency-scanning φ-OTDR. Opt. Lett..

[20] Sladen A (2019). Distributed sensing of earthquakes and ocean-solid Earth interactions on seafloor telecom cables. Nat. Commun..

[21] Walter F (2020). Distributed acoustic sensing of microseismic sources and wave propagation in glaciated terrain. Nat. Commun..

[22] Bashan G (2018). Optomechanical time-domain reflectometry. Nat. Commun..

[23] Chow DM (2018). Distributed forward Brillouin sensor based on local light phase recovery. Nat. Commun..

[24] Yan YX (2021). Distributed optical fiber sensing assisted by optical communication techniques. J. Lightwave Technol..

[25] Ip E (2022). Distributed fiber sensor network using telecom cables as sensing media: technology advancements and applications [Invited]. J. Opt. Commun. Netw..

[26] Marra G (2018). Ultrastable laser interferometry for earthquake detection with terrestrial and submarine cables. Science.

[27] Zhan ZW (2021). Optical polarization-based seismic and water wave sensing on transoceanic cables. Science.

[28] Mecozzi A (2021). Polarization sensing using submarine optical cables. Optica.

[29] Cantono, M. et al. Seismic sensing in submarine fiber cables. in *Proc. 2021 European Conference on Optical Communication*, 1–3. (IEEE, 2021).

[30] Mecozzi, A. Effects of geomagnetic field perturbations on the power supply of transoceanic fiber optic cables. Print at https://arxiv.org/abs/2204.00560 (2022) (preprint).

[31] Ip E (2022). Vibration detection and localization using modified digital coherent telecom transponders. J. Lightwave Technol..

[32] Zeng YF (2022). Integrated communication and polarization sensing in self-homodyne coherent systems. Opt. Lett..

[33] Lindsey NJ, Dawe TC, Ajo-Franklin JB (2019). Illuminating seafloor faults and ocean dynamics with dark fiber distributed acoustic sensing. Science.

[34] Williams EF (2019). Distributed sensing of microseisms and teleseisms with submarine dark fibers. Nat. Commun..

[35] Liu HY (2020). Vehicle detection and classification using distributed fiber optic acoustic sensing. IEEE Trans. Veh. Technol..

[36] Zhu TY, Shen JZ, Martin ER (2021). Sensing Earth and environment dynamics by telecommunication fiber-optic sensors: an urban experiment in Pennsylvania, USA. Solid Earth.

[37] Rodríguez Tribaldos V, Ajo-Franklin JB (2021). Aquifer monitoring using ambient seismic noise recorded with distributed acoustic sensing (DAS) deployed on dark fiber. J. Geophys. Res. Solid Earth.

[38] Huang, Y. K. & Ip, E. Simultaneous optical fiber sensing and mobile front-haul access over a passive optical network. in *Proc. 2020 Optical Fiber Communications Conference and Exhibition*. (IEEE, 2020).

[39] Huang MF (2020). First field trial of distributed fiber optical sensing and high-speed communication over an operational telecom network. J. Lightwave Technol..

[40] Guerrier, S. et al. Vibration detection and localization in buried fiber cable after 80km of SSMF using digital coherent sensing system with co-propagating 600Gb/s WDM channels. in *Proc. 2022 Optical Fiber Communications Conference and Exhibition*. (IEEE, 2022).

[41] Ip, E. et al. DAS over 1,007-km hybrid link with 10-Tb/s DP-16QAM co-propagation using frequency-diverse chirped pulses. in *Proc. 2022 Optical Fiber Communications Conference and Exhibition*, 1–3. (IEEE, 2022).

[42] Liu X (2013). Phase-conjugated twin waves for communication beyond the Kerr nonlinearity limit. Nat. Photonics.

[43] Gong XX (2017). SPM-improved transmission performance of software-reconfigurable IMDD PONs based on digital orthogonal filtering. J. Lightwave Technol..

[44] Chen HY (2015). High-capacity and high-loss-budget OFDM long-reach PON without an optical amplifier [Invited]. J. Opt. Commun. Netw..

[45] Jiang JL (2021). Continuous chirped-wave phase-sensitive optical time domain reflectometry. Opt. Lett..

[46] He HJ (2021). Suppression of the interference fading in phase-sensitive OTDR with phase-shift transform. J. Lightwave Technol..

[47] Chen D, Liu QW, He ZY (2017). Phase-detection distributed fiber-optic vibration sensor without fading-noise based on time-gated digital OFDR. Opt. Express.

[48] Qian H (2022). Fading-free Φ-OTDR evaluation based on the statistical analysis of phase hopping. Appl. Opt..

[49] Wang ZY (2015). Ultra-broadband phase-sensitive optical time-domain reflectometry with a temporally sequenced multi-frequency source. Opt. Lett..

[50] Chen D, Liu QW, He ZY (2018). High-fidelity distributed fiber-optic acoustic sensor with fading noise suppressed and sub-meter spatial resolution. Opt. Express.

[51] Loayssa A, Sagues M, Eyal A (2022). Phase noise effects on phase-sensitive OTDR sensors using optical pulse compression. J. Lightwave Technol..

[52] Xiong J (2021). High sensitivity and large measurable range distributed acoustic sensing with Rayleigh-enhanced fiber. Opt. Lett..

[53] Johansson LA, Seeds AJ (2003). Generation and transmission of millimeter-wave data-modulated optical signals using an optical injection phase-lock loop. J. Lightwave Technol..

[54] Lu B (2017). High spatial resolution phase-sensitive optical time domain reflectometer with a frequency-swept pulse. Opt. Lett..

[55] Pan Y (2019). Simultaneous demultiplexing of 2×PDM-PAM4 signals using simplified receiver. Opt. Express.

[56] Gardner FM (1986). A BPSK/QPSK timing-error detector for sampled receivers. IEEE Trans. Commun..

