# Supplementary material for: Integrated sensing and communication in an optical fibre
Source: Light Sci Appl. 2023 Jan 17;12:25. doi: 10.1038/s41377-022-01067-1 (PMC9845349; doi:10.1038/s41377-022-01067-1)
Supplement: Supplementary file 1 — Supplementary Information for Integrated Sensing and Communication in an Optical Fibre [file 41377_2022_1067_MOESM1_ESM.docx]

**Supplementary Information for Integrated Sensing and Communication in an Optical Fibre**

Haijun He^1^, Lin Jiang^1,2^, Yan Pan^1^, Anlin Yi^1^, Xihua Zou^1^, Wei Pan^1^, Alan E. Willner^3^, Xinyu Fan^4^, Zuyuan He^4^, Lianshan Yan^1,2^

^1^Center for Information Photonics & Communications, School of Information Science and Technology, Southwest Jiaotong University, Chengdu 611756, Sichuan, China

^2^Peng Cheng Laboratory, 518052, Shenzhen, China

^3^ Optical Fiber Communications Laboratory, University of Southern California, Los Angeles, California, 90089, USA

^4^ State Key Laboratory of Advanced Optical Communication Systems and Networks, Shanghai Jiao Tong University, 200240, Shanghai, China

Correspondence: Lianshan Yan

Email: [lsyan@home.swjtu.edu.cn](mailto:lsyan@home.swjtu.edu.cn)

**Theoretical analysis of the integrated sensing and communication in an optical fibre (ISAC-OF)**

In conventional PAM4 signal transmission based on external modulation, a single-frequency laser is served as the optical carrier. Different from the conventional PAM4 signal transmission, CW light with periodic linear frequency modulation (LFM) is utilized as the optical carrier in this work. For simplification, the optical field of the optical carrier is described as

$E\left(t\right)=A\exp\left[j\omega t+j2\pi f_{0}t+j\pi kt^{2}\right]$ (S1)

where *A* is the amplitude of the optical carrier, *ω* is the angular frequency of the output lightwave emitting from the light source, *f*_0_ is the offset frequency of the LFM signal, *k* is the chirp rate and equals to *B*/*T_p_*. *B* and *T_p_* are the bandwidth and repetition period of the LFM optical carrier, respectively. The illustration of the LFM-based CW light is depicted in Fig. S1.

**Figure S1.** The illustration of the periodic LFM optical carrier.

In conventional PAM4 transmission schemes, both *f*_0_ and *k* are equal to zero. Subsequenetly, the transmission code *X*(*t*) is applied to the Mach-Zehnder modulator (MZM) to generate the transmission light *E_X_*(*t*), as shown in Fig. S2.

**Figure S2.** Generation of the transmission light.

The optical field *E_X_*(*t*) is expressed as

 (S2)

In Eq. (S2), *x*(*t*) is the modulated phase generated by the transmission code and equals *πV_X_*(*t*)/*V_π_*, *V_X_*(*t*) is the signal voltage, *V_π_* is the half-wave voltage of MZM, *φ_V_* is the offset phase and it is determined by the bias voltage applied on the MZM. In PAM4 signal transmission, *φ_V_*=±*π*/2. With direct detection, the optical signal is converted into an electrical signal and expressed as

$I_{X}\left(t\right)=\Re\cdot E_{X}\left(t\right)\cdot E_{X}^{*}\left(t\right)=\frac{\Re A^{2}}{2}\left\{1\pm\sin\left[2x\left(t\right)\right]\right\}\approx\frac{\Re A^{2}}{2}\left[1\pm2x\left(t\right)\right]$ (S3)

In Eq. (S3), *ℜ* is the conversion coefficient of the photodetector, and |2*x*(*t*)| should be less than 0.4 to acquire undistorted code (i.e., |*x*(*t*)|≤0.2). It can be found that whether a single-frequency or an LFM optical carrier is used, the detected results are the same. Therefore, it is feasible to transmit the PAM4 signal using LFM optical carrier, and the demodulated method is the same as the conventional scheme. To clearly analyze the transmission and sensing performances, the code-related function *A*cos[*x*(*t*)+*φ_V_*/2] in Eq. (S2) is expanded. Meanwhile, the offset phase *φ_V_*=*π*/2 is substituted into Eq. (S2).

 (S4)

Since the amplitude of the transmission code is very small (|*x*(*t*)|≤0.2), cos[*x*(*t*)]-sin[*x*(*t*)] can be simplified by Taylor expansion, and Eq. (S4) is rewritten as

$E_{X}\left(t\right)\approx\frac{\sqrt{2}}{2}A\left[1-\frac{x^{2}\left(t\right)}{2}-x\left(t\right)\right]\exp\left(j\omega t+j2\pi f_{0}t+j\pi kt^{2}+j\frac{\pi}{4}\right)$ (S5)

For ease of analysis, *E_X_*(*t*) in Eq. (S5) is decomposed into two parts (carrier- and code-related functions) and expressed as

$E_{X}\left(t\right)=E_{dc}\left(t\right)+E_{ac}\left(t\right)$ (S6)

$E_{dc}\left(t\right)=\frac{\sqrt{2}}{2}A\exp\left(j\omega t+j2\pi f_{0}t+j\pi kt^{2}+j\frac{\pi}{4}\right)$ (S7)

$E_{ac}\left(t\right)=-\frac{\sqrt{2}}{2}A\left[\frac{x^{2}\left(t\right)}{2}+x\left(t\right)\right]\exp\left(j\omega t+j2\pi f_{0}t+j\pi kt^{2}+j\frac{\pi}{4}\right)$ (S8)

where *E_dc_*(*t*) is a pure optical carrier, *E_ac_*(*t*) is the code-related lightwave. The power of *E_dc_*(*t*) is much larger than the power of *E_ac_*(*t*) in PAM4 signal transmission, over 16 dB generally. Hence, the weak DC component $-\sqrt{2}/4A\overline{x^{2}\left(t\right)}$ is not split from *E_ac_*(*t*). When a large launching power is employed in the conventional PAM4 transmission (*ω*_0_=0, *k*=0), an intensive SBS is induced by the strong optical carrier. That distorts the transmission code and deteriorates the BER performance. In order to achieve good BER performance, the carrier power of the transmission light is limited below the SBS threshold (weak SBS interaction and weak Stokes light are allowed). Different from the conventional scheme, an LFM optical carrier is used in the proposed scheme, and it effectively suppresses the SBS interaction. Benefitting from this property, both launching power and transmission performance can be significantly improved.

When the transmission light *E_X_*(*t*) is launched into the fibre, the corresponding Rayleigh backscattering (RBS) light *E_R_*(*t*) is generated and expressed as

$\begin{matrix} E_{R}\left(t\right)=\sum_{i=1}^{N} \frac{\sqrt{2}}{2}a\left(\tau_{i}\right)r\left(\tau_{i}\right)\cdot A\left[1-\frac{x^{2}\left(t-\tau_{i}\right)}{2}-x\left(t-\tau_{i}\right)\right] \\ \cdot\exp\left[j\omega\left(t-\tau_{i}\right)+j2\pi f_{0}\left(t-\tau_{i}\right)+j\pi k\left(t-\tau_{i}\right)^{2}+j\frac{\pi}{4}\right] \\ =\sum_{i=1}^{N} a\left(\tau_{i}\right)r\left(\tau_{i}\right)E_{X}\left(t-\tau_{i}\right) \end{matrix}$ (S9)

where *τ_i_* is the round-trip time of the lightwave traveling from the near end to the *i*-th reflector, *a*(*τ_i_*) is the attenuation coefficient, *r*(*τ_i_*) is the Rayleigh scattering coefficient of the *i*-th reflector, and *r*(*τ_i_*)=|*r*(*τ_i_*)|exp[*jϕ*(*τ_i_*)], *ϕ*(*τ_i_*) is the random phase of the *i*-th reflector, *N* is the total number of Rayleigh reflectors in a fibre.

In sensing signal detection, a coherent receiver is employed to acquire the quadrature signals and eliminate the polarization-dependent fading. The RBS light *E_R_*(*t*) is decomposed into two orthogonal-polarization components.

$E_{RX}\left(t\right)=\sum_{i=1}^{N} a\left(\tau_{i}\right)r\left(\tau_{i}\right)\cos\left[\theta\left(\tau_{i}\right)\right]E_{X}\left(t-\tau_{i}\right)$ (S10)

$E_{RY}\left(t\right)=\sum_{i=1}^{N} a\left(\tau_{i}\right)r\left(\tau_{i}\right)\sin\left[\theta\left(\tau_{i}\right)\right]E_{X}\left(t-\tau_{i}\right)$ (S11)

In Eq. (S10) and Eq. (S11), *θ*(*τ_i_*) is the relative angle between the RBS light and the local oscillator (LO), cos[*θ*(*τ_i_*)] and sin[*θ*(*τ_i_*)] are the decomposition coefficients in the *X*- and *Y*-polarization states, respectively. Assuming that LO is *E_L_*(*t*)=*A_L_*exp[*jωt*+*j*2*πf*_1_*t*], four electrical signals *i_IX_*(*t*), *i_QX_*(*t*), *i_IY_*(*t*), and *i_QY_*(*t*) are collected at the coherent receiver. By combining two quadrature signals at the same polarization state, the complex RBS fields (in *X*- and *Y*- polarization states) are obtained and described as

$\begin{matrix} I_{RX}\left(t\right)=i_{IX}\left(t\right)+j\cdot i_{QX}\left(t\right) \\ =real\left[\Re_{s}\cdot E_{RX}\left(t\right)\cdot E_{L}^{*}\left(t\right)\right]+j\cdot imag\left[\Re_{s}\cdot E_{RX}\left(t\right)\cdot E_{L}^{*}\left(t\right)\right] \\ =\Re_{s}\cdot\left\{s\left(t\right)\otimes\left[\cos\left(\theta\left(t\right)\right)\cdot h\left(t\right)\right]\right\} \end{matrix}$ (S12)

$\begin{matrix} I_{RY}\left(t\right)=i_{IY}\left(t\right)+j\cdot i_{QY}\left(t\right) \\ =real\left[\Re_{s}\cdot E_{RY}\left(t\right)\cdot E_{L}^{*}\left(t\right)\right]+j\cdot imag\left[\Re_{s}\cdot E_{RY}\left(t\right)\cdot E_{L}^{*}\left(t\right)\right] \\ =\Re_{s}\cdot\left\{s\left(t\right)\otimes\left[\sin\left(\theta\left(t\right)\right)\cdot h\left(t\right)\right]\right\} \end{matrix}$ (S13)

where *real*[ ] and *imag*[ ] represent the real and imaginary parts respectively. *ℜ_s_* is the conversion coefficient of the coherent receiver, * denotes the conjugate symbol, is the convolution operator. *s*(*t*) is the equivalent light field, *h*(*t*) is the impulse response of the fibre. *s*(*t*) and *h*(*t*) are expressed as

$\begin{matrix} s\left(t\right)=\frac{\sqrt{2}}{2}AA_{L}\left[1-\frac{x^{2}\left(t\right)}{2}-x\left(t\right)\right]\exp\left[j2\pi\left(f_{0}-f_{1}\right)t+j\pi kt^{2}+j\frac{\pi}{4}\right] \\ h\left(t\right)=a\left(t\right)r\left(t\right)\exp\left(-j\omega t-j2\pi f_{1}t\right) \end{matrix}$ (S14)

It can be seen that the equivalent light field *s*(*t*) is highly similar to the transmission light *E_X_*(*t*). Similar to Eqs. (S6)-(S8), *s*(*t*) is divided into two parts and expressed as

$s\left(t\right)=s_{dc}\left(t\right)+s_{ac}\left(t\right)$ (S15)

$s_{dc}\left(t\right)=\frac{\sqrt{2}}{2}AA_{L}\exp\left[j2\pi\left(f_{0}-f_{1}\right)t+j\pi kt^{2}+j\frac{\pi}{4}\right]$ (S16)

$s_{ac}\left(t\right)=-\frac{\sqrt{2}}{2}AA_{L}\left(\frac{x^{2}\left(t\right)}{2}+x\left(t\right)\right)\exp\left[j2\pi\left(f_{0}-f_{1}\right)t+j\pi kt^{2}+j\frac{\pi}{4}\right]$ (S17)

In above expressions, *s_dc_*(*t*) is the beat signal of *E_dc_*(*t*) and the local light *E_L_*(*t*), *s_ac_*(*t*) is the beat signal of *E_ac_*(*t*) and *E_L_*(*t*). Note that the power ratio between *s_dc_*(*t*) and *s_ac_*(*t*) is the same as the power ratio between *E_dc_*(*t*) and *E_ac_*(*t*).

**Figure S3.** Sensing signal demodulation in the ISAC-OF.

In the signal demodulation, a digital matched filter (DMF) is generated in the digital domain. By convoluting the signals of *I_RX_*(*t*) and *I_RY_*(*t*) with the DMF *s_dc_**(-*t*), as depicted in Fig. S3, the effective RBS signals are retrieved and expressed as

$\begin{matrix} r_{X}\left(t\right)=I_{RX}\left(t\right)\otimes s_{dc}^{*}\left(-t\right) \\ =\Re_{s}\cdot\left\{\left[\cos\left(\theta\left(t\right)\right)\cdot h\left(t\right)\right]\otimes R\left(t\right)\right\}+\Re_{s}\cdot\left\{\left[\cos\left(\theta\left(t\right)\right)\cdot h\left(t\right)\right]\otimes n\left(t\right)\right\} \end{matrix}$ (S18)

$\begin{matrix} r_{Y}\left(t\right)=I_{RY}\left(t\right)\otimes s_{dc}^{*}\left(-t\right) \\ =\Re_{s}\cdot\left\{\left[\sin\left(\theta\left(t\right)\right)\cdot h\left(t\right)\right]\otimes R\left(t\right)\right\}+\Re_{s}\cdot\left\{\left[\sin\left(\theta\left(t\right)\right)\cdot h\left(t\right)\right]\otimes n\left(t\right)\right\} \end{matrix}$ (S19)

where

$R\left(t\right)=s_{dc}\left(t\right)\otimes s_{dc}^{*}\left(-t\right)$ (S20)

$n\left(t\right)=s_{ac}\left(t\right)\otimes s_{dc}^{*}\left(-t\right)$ (S21)

*R*(*t*) is the equivalent pulse probe, its pulse duration is determined by the bandwidth of the LFM optical carrier. *n*(*t*) is the convolution result between *s_ac_*(*t*) and *s_dc_**(-*t*), it is detrimental to the sensing performance. The signal-to-noise ratio (SNR) of the effective RBS signal is determined by the power ratio between *R*(*t*) and *n*(*t*), which equals the power ratio between *s_dc_*(*t*) and *s_ac_*(*t*). Notice that the power ratio between *s_dc_*(*t*) and *s_ac_*(*t*) is equal to the power ratio between *E_dc_*(*t*) and *E_ac_*(*t*). Therefore, the power of the effective RBS signal (demodulating with *r_X_*(*t*) or *r_Y_*(*t*)) and noise is proportional to the power of *E_dc_*(*t*) and *E_ac_*(*t*), respectively. Since the power of *E_ac_*(*t*) is much smaller than *E_dc_*(*t*), the sensing performance is only slightly degraded.

**Estimation of sensing performance**

In principle, the sensing performance of a system is mainly determined by SNR. Thus, we calculate the SNR of the integrated scheme (*SNR_i_*) to estimate the sensing performance. *SNR_i_* is expressed as

$\begin{matrix} SNR_{i}=\frac{\left\langle I_{dc}^{2}\right\rangle}{\left\langle I_{ac}^{2}\right\rangle+\sigma^{2}}=\frac{\frac{1}{2}\Re_{s}^{2}rP_{dc}P_{L}}{\frac{1}{2}\Re_{s}^{2}rP_{ac}P_{L}+\frac{1}{2}q\left[\Re_{s}\left(P_{L}+rP_{dc}+rP_{ac}\right)+4I_{d}\right]\Delta f+\sigma_{T}^{2}} \\ \approx\frac{1}{\frac{P_{ac}}{P_{dc}}+\left(\frac{1}{rP_{dc}}+\frac{1}{P_{L}}\right)\frac{q\Delta f}{\Re_{s}}+\frac{4qI_{d}\Delta f+2\sigma_{T}^{2}}{rP_{dc}P_{L}\Re_{s}^{2}}} \end{matrix}$ (S22)

where *P_dc_*, *P_ac_*, and *P_L_* denote the average power of *E_dc_*(*t*), *E_ac_*(*t*), and *E_L_*(*t*), respctively. *r* is the RBS coefficient, *q* is the electron, Δ*f* is the receiver bandwidth, *I_d_* is the dark current of detector, $\sigma_{T}^{2}$ is the thermal noise.

Besides, the SNR of the pure LFM-based Φ-OTDR (*SNR_s_*) is also computed to compare with the SNR in the integrated scheme. Assume that the same probe power *P_dc_* as the integrated scheme is adopted, *SNR_s_* is

$SNR_{s}=\frac{\left\langle I_{dc}^{2}\right\rangle}{\sigma^{2}}=\frac{1}{\left(\frac{1}{rP_{dc}}+\frac{1}{P_{L}}\right)\frac{q\Delta f}{\Re_{s}}+\frac{4qI_{d}\Delta f+2\sigma_{T}^{2}}{rP_{dc}P_{L}\Re_{s}^{2}}}$ (S23)

By comparing Eq. (S22) and Eq. (S23), we can find that the perfromance degradation is determined by the power ratio *P_ac_*/*P_dc_*, which is the inverse of the carrier-sideband ratio (*CSR*).

$CSR=\frac{P\left[E_{dc}\left(t\right)\right]}{P\left[E_{ac}\left(t\right)\right]}=\frac{1}{\left\langle x^{2}\left(t\right)+x^{3}\left(t\right)+\frac{x^{4}\left(t\right)}{4}\right\rangle}\approx\frac{9}{5A_{m}^{2}}$ (S24)

where *P*[*E_dc_*(*t*)] and *P*[*E_ac_*(*t*)] represent the average power of the DC and AC components, respectively. *A_m_* is the maximum value of the PAM4 signal and it is less than 0.2 generally (i.e., *CSR*>45). If *P_L_* and *P_dc_* remain unchanged, *SNR_i_* improves with increasing *CSR*. Theoretically, *SNR_i_* is proportional to *P_L_* and *P_dc_* when *CSR* is kept unchanged. However, the coefficient *q*Δ*f*/*ℜ_s_* is too small, resulting in a negligible change in *SNR_i_*. Therefore, the sensing performance should remain the same for a fixed *CSR* regardless of whether the launching power is increased or decreased.

Usually, the code bandwidth is larger than the carrier bandwidth (i.e., the bandwidth of the sensing probe). So, the sensing performance can be improved by removing the out-of-carrier-band noise in signal demodulation using an appropriate digital filter. Assuming that the bandwidth of the digital filter is equal to the carrier bandwidth, the effective *CSR* should be corrected using the bandwidth ratio *R_B_* between *E_ac_*(*t*) and *E_dc_*(*t*). Here, *R_B_*=*B*[*E_ac_*(*t*)]/*B*[*E_dc_*(*t*)], the corrected *CSR* (*CSR_c_*) is expressed as

$CSR_{c}=CSR\cdot R_{B}=\frac{9}{5A_{m}^{2}}\cdot\frac{B\left[E_{ac}\left(t\right)\right]}{B\left[E_{dc}\left(t\right)\right]}$ (S25)

For a fixed launching power, both the effective *CSR* and the sensing performance improve with increasing baud rate or decreasing LFM bandwidth. By dividing Eq. (S22) and Eq. (S23), the accurate SNR degradation can be calculated.

$\frac{SNR_{i}}{SNR_{s}}=\frac{1}{\frac{SNR_{s}}{CSR_{c}}+1}$ (S26)

In experiments, the bandiwdth of 28-Gbaud signal is about 15 GHz due to using shaped filtering in the signal generation. The effective CSR is equal to $\frac{27}{2A_{m}^{2}}$ for 28-Gbaud transmission code and 2-GHz LFM carrier. Assuming that *SNR_s_* is 25 dB (~316 in linear unit), the SNR is degraded less than 1 dB and 3 dB if *A_m_* is equal to 0.1 and 0.2, respectively. According to the above analysis, it can be found that the theoretical degradation of the sensing performance in the proposed integration scheme is very small, and it is acceptable in practical applications.

**Transmission decoding in the ISAC-OF**

The decoding algorithm is the same as the conventional PAM4 signal transmission and the decoding procedure is depicted in Fig. S4. The decoding algorithm is mainly divided into five steps. 1) Resample. The signals are resampled to 4 Sa/symbol. 2) Matched filtering. The signals are filtered with a matched filter to recover the effective signals. 3) Clock recovery and downsample. Gardner’s method is used to recover the clock and get the optimal sampling point and then downsample the signals to 1 Sa/symbol for further processing. 4) Equalization. The channel equalization is implemented with the decision-directed least mean square error algorithm (DD-LMS). 5) Decision and BER calculation.

**Figure S4.** The algorithm procedure of the PAM4 decoding.
